# Field Network—A New Method to Detect Directional Object

**DOI:** 10.3390/s20154262

**Published:** 2020-07-30

**Authors:** Jin Liu, Yongjian Gao

**Affiliations:** State Key Laboratory of Information Engineering in Surveying, Mapping and Remote Sensing, Wuhan University, Wuhan 430079, China; jliu@sgg.whu.edu.cn

**Keywords:** field network, object detection, direction prediction

## Abstract

As the development of object detection technology in computer vision, identifying objects is always an active yet challenging task, and even more efficient and accurate requirements are being imposed on state-of-the-art algorithms. However, many algorithms perform object box regression based on RPN(Region Proposal Network) and anchors, which cannot accurately describe the shape information of the object. In this paper, we propose a new object detection method called Field Network (FN) and Region Fitting Algorithm (RFA). It can solve these problems by Center Field. Center field reflects the probability of the pixel approaching the object center. Different from the previous methods, we abandoned anchors and ROI technologies, and propose the concept of Field. Field is the intensity of the object area, reflecting the probability of the object in the area. Based on the distribution of the probability density of the object center in the visual field perception area, we add the Object Field in the output part. And we abstract it into an Elliptic Field with normal distribution and use RFA to fit objects. Additionally, we add two fields to predict the x,y components of the object direction which contain the neural units in the field array. We extract the objects through these Fields. Moreover, our model is relatively simple and have smaller size, which is only 73 M. Our method improves performance considerably over baseline systems on DOTA, MS COCO and PASCAL VOC datasets, with overall performance competitive with recent state-of-the-art systems.

## 1. Introduction

Owing to the continual development of computer vision technology in recent years, object detection has entered a new era [1,2,3]. However, we also have to face the complexity and cost of the resources [2]. These problems have been around for a long time and attracted much attention in the past decade [4,5,6,7].

Traditional two-stage algorithms mainly train two parts. The first step is to train the RPN(Region Proposal Network) network, and the second step is to train the network of object area detection [2,4,5,8]. Compared with one-stage algorithms, their network has high accuracy but relatively slow speed. On the other hand, one-stage algorithms are often fast but not accurate enough [1,3,9,10,11,12,13]. Although there are some algorithms that take both speed and accuracy into account, they are not satisfactory because they lack sufficient depth of semantic information [10,14,15,16,17,18,19].

In the experiment, we discover that when the grid density is large, the convolution network’s ability to express the intensity of the object area will be improved correspondingly, but the ability to express the spatial information of the object will be reduced. The dense output means that the same length information representing the object needs to span more neurons. Since the single-layer convolution operation spans a limited number of neurons, this requires a deeper convolutional layer network to support, while deeper networks require more feature maps. Therefore, when the output density increases, a large model is needed to support the coordinate regression of high precision object position.

Traditional algorithms do not have enough ability to directly describe the coordinate position of the object. Moreover, these algorithms use techniques such as anchors, NMS and ROI (Non-Maximum Suppression and Region of Interest)pooling [2,8,20,21]. However, these techniques are based on the horizontal recommendation box of RPN, and the object shape and direction are various, and contains many invalid areas. Meanwhile, semantic segmentation has strong learning ability for pixel-by-pixel classification and does not require very large models to support coordinate regression of high-precision object positions [5,6,22,23,24]. However, the classification of each pixel of semantic segmentation is isolated, and the same type of object will be connected [7].

**Figure 1:**
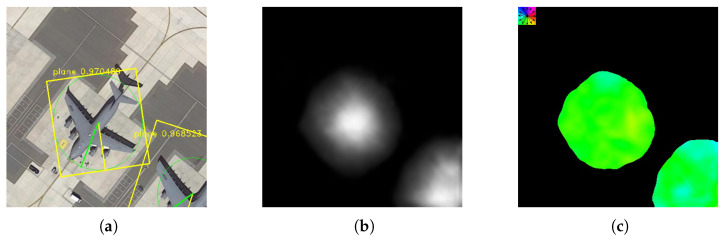
The presentation of object field. (**a**) is original image, (**b**) is Center Field and (**c**) is Direction Field. We can fit the object by (**b**). Also, we can get the direction through (**c**).

To solve the aforementioned problems, in this paper we propose a new object detection model called Field Network (FN). Field is the intensity of the object area, reflecting the probability of the object in the area. The field is shown in Figure 1. We combine the advantages of object detection and semantics segmentation, effectively avoid their respective shortcomings, so that the detection speed and accuracy are greatly improved. Moreover, when we add a direction field to the object field, we can also get the direction. We choose to regress the direction vector instead of the direction angle to obtain the object direction. This is because the regression direction angle will have the angle circulation problem, for example, there is a considerable error between θ and θ+2π. And we extensively test and evaluate the FN algorithm on three public datasets for object detection in References [25,26,27], and compare it with state-of-the-art methods.

The main contributions of this paper are as follows.

- We propose the concept of Field. Based on the Field, our framework can distinguish the overlapping regions of the same object on the basis of Center Field. From this we can get the center coordinates, the range of the area, and the total number of objects for each one.
- We design a Field-based object Region Fitting Algorithm (RFA), which abandons some traditional techniques and makes the algorithm efficient and accurate for object detection.
- We can also get the direction of the object through the Direction Field by regressing the direction vector.

## 2. Related Work

Recent years have witnessed a vast amount of work on the computer vision. Among them, the fastest growing tasks can be divided into two classical categories—object detection and object segmentation.

The first category of popular object detection algorithms can be divided into two categories, two-stage and one-stage [1,2,3,4,5,7,8,9,10,14,28]. RCNN(Regions with CNN features) [4] is the pioneering of the two-stage algorithm. It used a convolutional neural network (CNN) for the first time in the field of object detection, which greatly improved the effect of target detection. After several years of development, CNNs showed its strong vitality. The most representative of these is Faster-RCNN [2]. It generates region proposals from the RPN network and then classifies the regions proposals. It greatly improves the accuracy of object detection, but at the same time its speed is relatively unsatisfactory. After obtaining the region proposals, the calculation amount for each proposal classification is still relatively large. This affects its computational efficiency to some extent. The one-stage algorithm [1,3,9,10,14] is region-free, which converts the problem of object detection into a regression problem, but the speed is improved and the accuracy is not enough. Our method also discards the region proposal, and instead proposed the concept of Object Field, which can balance accuracy and efficiency.

Another type of algorithm is called object segmentation and the pioneering is FCN(Fully Convolutional Networks). What FCN [6] pursues is that the input is a picture, and the output is also a picture. It proposes a full convolutional neural network and learns pixel to pixel mapping and end-to-end mapping. The full convolutional network mainly uses three techniques, convolutional, upsample and skip layer. But there are still many problems that cannot be avoided, such as accuracy problems, insensitivity to details and ignoring spatial consistency, and so forth. U-Net [22] is used to solve simple problem segmentation of small samples. It is improved on the basis of FCN. U-Net uses excessive data augmentation by applying elastic deformations to the available training images, to some extent solves the problem of too few samples in some scenarios. Our algorithm uses it as a backbone, adds Object Field to the output, and then uses a fitting algorithm to detect the object.

## 3. Algorithm

### 3.1. Object Field

The convolutional neural network can be abstracted into a mathematical model Y = F (W, X), where X is the input, Y is the output, and W is the convolution kernel parameter. CNN can be seen as a directed acyclic graph from X to Y. Its basic architecture consists of input layer, convolutional layer, pooling layer, upsample layer and output layer. Therefore, when designing the network structure F, it should be able to express Y more quickly and accurately. However, in the CNNs, the pooling layer extracts the intensity information of the object, and the spatial information, such as the maximum response neuron offset coordinate and the object width and height, cannot be transmitted by the pooling layer. Therefore, this convolutional network has a weak ability to express spatial information. So in order for the convolutional network to better express spatial information, we add an object output field to regress the probability of the objects Y appearing in the image. Because the values of the central field data is in the range of [0, 1], we transform the output layer of the neural network into the final field output value through the logistic activation function. Through the object output field, we can further obtain the location information.

The object output field is the probability distribution map of the object appearing on the image. The probability of the object center is the largest, and the closer to the edge, the lower the probability. This field can be expressed by the two-dimensional normal distribution formula:

(1) $$\left\{\begin{array}{c} f\left(x,y\right)=\frac{1}{2\pi \sigma_{1}\sigma_{2}\sqrt{1-\rho^{2}}}e^{-k}\\ k=\frac{1}{2\left(1-\rho^{2}\right)}\left[\frac{{\left(x-\mu_{1}\right)}^{2}}{\sigma_{1}^{2}}-2\rho \frac{x-\mu_{1}}{\sigma_{1}}\cdot \frac{y-\mu_{2}}{\sigma_{2}}+\frac{{\left(y-\mu_{2}\right)}^{2}}{\sigma_{2}^{2}}\right]. \end{array}\right.$$

We can get a maximum probability when the field coordinates are at the center of the ellipse. According to this definition, we use neural networks to regress this field probability information in an elliptical distribution.

**Figure 2:**
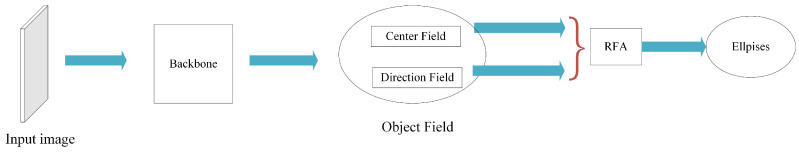
The architecture of Field Network. We add an elliptical field to the output, which contains the Center Field and the Direction Field. The two fields respectively output C feature maps corresponding to the regional distribution field of the C-type object.

On the basis of backbone, we added the object field to the output section. We abstract the object field into a normally distributed elliptical field containing two components, the Center Field and the Edge Field. The architecture is shown in Figure 2. We give the loss function as

(2) $$loss=\lambda_{s}{loss}_{softmax}+\lambda_{c}{loss}_{\mathrm{center}}+\lambda_{d}{loss}_{direction},$$

where the $loss_{\mathrm{center}},loss_{direction}$ are defined in Equations (5) and (6) respectively.

**Figure 3:**
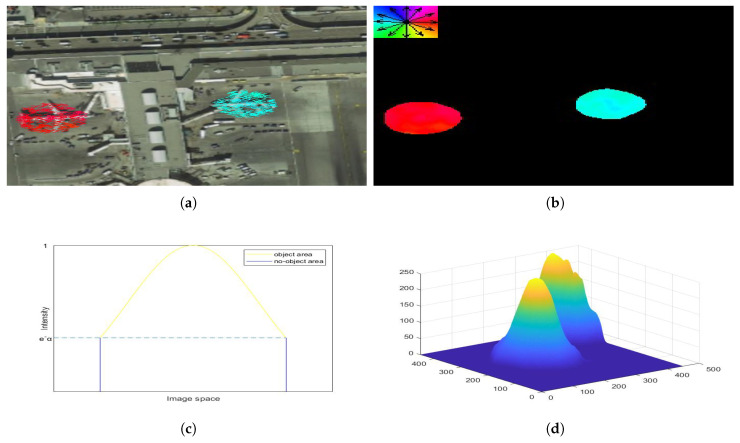
Intensity distribution of object in the field. (**a**) is the direction diagram, (**b**) is the Direction Field. (**c**) is the two-dimensional graps of the Center Field. (**d**) is the three-dimensional graph of the Center Field.

Center Field. The intensity of the normal distribution is related to the elliptic equation, so we use the elliptic equation to describe the distribution of an object on a two-dimensional image. The output value of the center field indicates the probability that the pixel is close to the target center, so we define the range of values for each output element to be [0, 1]. The output intensity of each pixel is calculated by

(3) $$G_{ccp}=\left\{\begin{array}{cc} e^{-\alpha d_{cpi}^{2}} & d_{cpi}^{2}\leq 1\\ 0 & \;\mathrm{other}\; \end{array}\right.,$$

where ’ccp’ is an abbreviation of center class pixel. And $G_{ccp}$ is the ground truth of the pixel P of feature map of class C in the center field. $d_{cpi}$ is the distance from the pixel P in the class C feature map to the i-th object. Figure 3 shows the distribution of object intensity in image space. To build this mathematical model, we describe how close the pixel is to the center of the object by

(4) $$\left\{\begin{array}{c} {d_{i}}^{2}=\frac{{\left(\cos\theta \cdot dx_{i}+\sin\theta \cdot dy_{i}\right)}^{2}}{a^{2}}+\frac{{\left(-\sin\theta \cdot dx_{i}+\cos\theta \cdot dy_{i}\right)}^{2}}{b^{2}}\leq 1\\ d_{x_{i}}=x_{i}-x_{0}\\ d_{y_{i}}=y_{i}-y_{0} \end{array}\right.,$$

and we give the loss function of Center Field as

(5) $${loss}_{center}=\sum_{c=1}^{C}\sum_{p\in field}{\left(v_{ccp}-G_{ccp}\right)}^{2}.$$

Direction Field. The Direction Field is used to describe the direction information of the object and requires the training dataset to have direction information. We also add 2×C channels to output the x,y direction field of the *C* class object, then the direction vector of a certain neuron in the field is $\left\{Q_{0}\left(x,y\right),Q_{1}\left(x,y\right)\right\}$. The loss function of Direction Field is

(6) $${loss}_{dir}=\sum_{c}w_{c}\sum_{x,y}\delta \left(c\right)E_{xy},$$

where $w_{c}=1$ if an object has diectioin and $w_{c}=0$ if not. Meanwhile, if x,y belongs to at least one object in the field, then δ(c)=1, otherwise δ(c)=0. We give $E_{xy}$ as

(7) $$E_{xy}={\left(v_{dxcp}-G_{dxcp}\right)}^{2}+{\left(v_{dycp}-G_{dycp}\right)}^{2},$$

where $G_{dxcp}$ and $G_{dycp}$ are ground truth of the x and y component of the Direction Field at the object point p of class c respectively.

We define the default value of the back propagation weight of the Direction Field $\lambda_{d}=2$ in Equation (2). According to the theory of constrained neural networks, we unitize $Q_{0}$ and $Q_{1}$ to obtain the direction vector $q_{0}$ and $q_{1}$ at x,y by

(8) $$\left\{\begin{array}{c} q_{0}\left(x,y\right)=\frac{Q_{0}\left(x,y\right)}{L}\\ q_{1}\left(x,y\right)=\frac{Q_{1}\left(x,y\right)}{L}\\ L=\sqrt{Q_{0}^{2}\left(x,y\right)+Q_{1}^{2}\left(x,y\right)} \end{array}\right..$$

**Figure 4:**
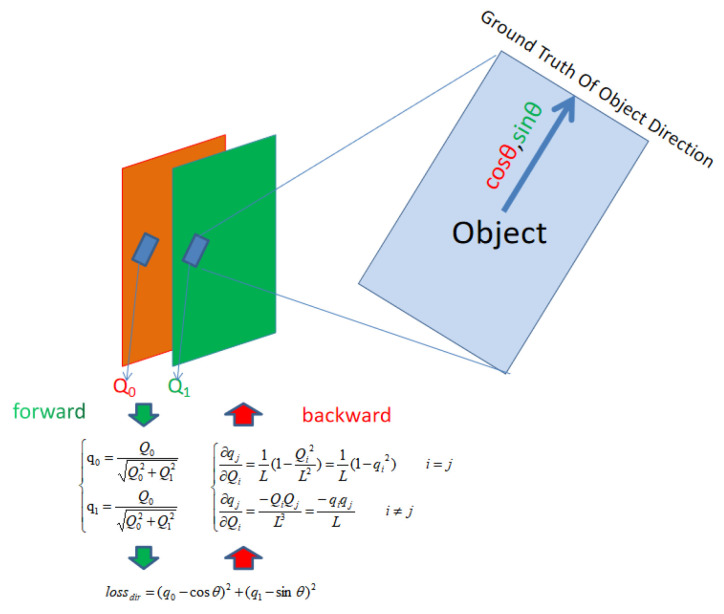
Forward and backward of the object direction calculation.

The object direction represented by rotation angle in regression will lead to the ambiguity of direction. To solve this problem, we use the unitization constraint algorithm to obtain the object unitization direction vector $\left\{q_{0},q_{1}\right\}$, which is used to regress to the Ground Truth direction of the object. As shown in Figure 4, the output values of the two channels of the direction output layer of a neuron in the object area $\left\{Q_{0},Q_{1}\right\}$ are converted to $\left\{q_{0},q_{1}\right\}$ by unitization.

The direction of each iron atom in the magnet determines the direction of the magnetic field. For the same principle, we find the direction of each point in the Direction Field in the RFA to get the direction of the object. Then we can calculate the direction of the object by

(9) $$d_{object}=\frac{\frac{1}{n}\sum_{j=1}^{n}\left\{q_{j0},q_{j1}\right\}}{\left|\frac{1}{n}\sum_{j=1}^{n}\left\{q_{j0},q_{j1}\right\}\right|},$$

which is used to calculate the average direction of n points in the object area. The detailed description of the object points searching can be found in Section 3.2.

In the DOTA [25] dataset, the object is described by four clockwise enclosing points $P_{0},P_{1},P_{2},P_{3}$, where $P_{0}$ is the left front point relative to the object itself. The front end center point $P_{f}$ and the back end center point $P_{b}$ of the object can be obtained by

(10) $$\left\{\begin{array}{c} P_{f}=\frac{1}{2}\left(P_{0}+P_{1}\right)\\ P_{b}=\frac{1}{2}\left(P_{2}+P_{3}\right) \end{array}\right.,$$

then we can get main direction of the object by

(11) $$\left(G_{dxcp},G_{dycp}\right)=\frac{P_{f}-P_{b}}{\left|P_{f}-P_{b}\right|}.$$

**Figure 5:**
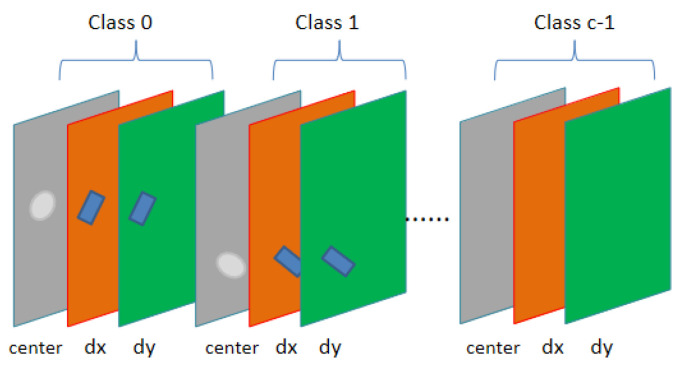
3c feature maps for c classes object field regression.

Figure 5 shows the number and composition of the feature maps of the output layer, where c is the number of classes. In order to represent the two-dimensional direction, we output two direction fields as well as the centers field.

### 3.2. Region Fitting Algorithm

In this section, we propose a field-based object region fitting algorithm called RFA. We process the Center Field and the Direction Field. The output feature maps of the Center Field and the Direction Field are C and 2 × C respectively, which represent class C objects and 2 × C direction vectors. At inference, we choose the largest field of pixel P in the output C-class Center Fields to get the class of P.

Getting the object point according to the Center Field. For the output value of each pixel in the center field, if $value\geq e^{-\alpha }$, search for the maximum intensity value that has not been searched in the eight neighborhoods of the pixel. Then move to the position of this maximum value and repeat the search step until there is no greater value around it, then note the coordinates of the point $\left(x_{c},y_{c}\right)$.

Getting the object edge point sets by searching the Center Field from the center point $\left(x_{c},y_{c}\right)$. We use the center point as the starting point to get the point set of the edge area of each object through breadth-first search. As shown in Figure 3b, we spiral down from top to bottom to search the entire Center Field. The whole search process is as follows:

**Step 1:** Initializing a queue Q and put the starting point $P_{0}$ into Q.

**Step 2:** The head element $P_{i}$ in Q is taken out, and then the 8 pixel neighborhood points $P_{k}\left\{k=1,2,3\ldots 8\right\}$ of $P_{i}$ are pushed into Q by value $V_{k}$ in Center Field in descending order. $P_{k}$ must be a point that has not been searched.

**Step 3:** Repeat **Step 2** util Q is empty. In addition, if the average intensity of all points in Q is less than 0.5, the loop is exited. Finally, we can get all the point sets $\left\{x_{j},y_{j},v_{j}\right\}$ in Q corresponding to the starting point $P_{0}$.

**Step 4:** We sample the point set in the Direction Field to get $Q_{0},Q_{1}$ in the object, then get the unitized vector $q_{0},q_{1}$ of each point by Equation (8), and then get the whole direction vector according to Equation (9).

**Figure 6:**
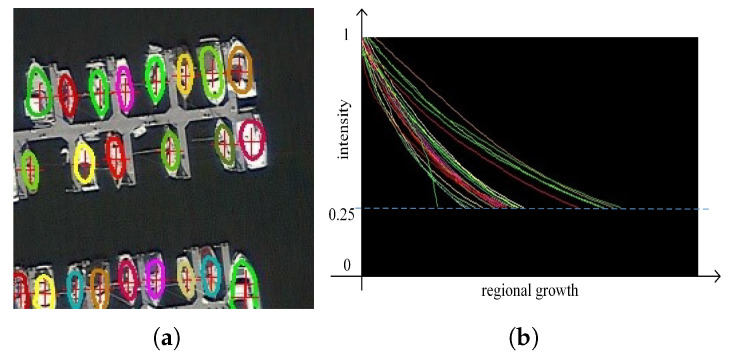
Visual demonstration of Region Fitting Algorithm (RFA) algorithm. (**a**) is a graph of candidate edge points superimposed with multiple objects. (**b**) is the average intensity of the points in the queue during regional growth, and each of the different colored curves corresponds to an object in (**a**).

Figure 6 is a diagram illustrating the above algorithm. It can be seen from the Figure 6b that as the iteration progresses, the search range gradually expands, and the center field strength of each object gradually decreases during the regional growth iteration process. When the average value is around 0.25, the center field intensity tends to be flat and there is a sufficient amount of sampled data. At this time, ellipse fitting can be performed, and the region growth process of a single object ends. This algorithm can converge quickly, and can collect a sufficient number of points that can regress to the object ellipse parameters.

Calculating the elliptic equation of the object. An ellipse can effectively describe the regional distribution of an object of arbitrary aspect ratio in the image space. We substitute the edge points into Equation (4), and use the LM(Levenberg-Marquard) algorithm to solve the equations. In addition, we add a central restraint condition as

(12) $$\lambda \left(x_{0}^{2}+y_{0}^{2}\right)=0.$$

Since the value interval of the center point $\left(x_{0},y_{0}\right)$ is [0, 1], we define the default value α=2000 to have a better effect. According to Equations (3) and (4), we can get $d_{i}^{2}=-\frac{\log\left(Y_{cpi}\right)}{\alpha }$, where $Y_{cpi}$ is the output of the neural network at the pixel $p_{i}$ of the Center Field. Then we give the Jacobian matrix equations as shown in Equation (13). Where a and b are the major axis and minor axis of the ellipse respectively, and θ is the inclination angle of the ellipse. Because the ellipse is symmetric, the exact direction of the object needs to be further determined by the direction field. $x_{0}$ and $y_{0}$ are the offset of the ellipse from the search center. And $F_{i}$ is the value of the ellipse field at pixel $p_{i}$.

(13) $$\left[\begin{array}{ccccc} \frac{\partial F_{1}}{\partial a} & \frac{\partial F_{1}}{\partial b} & \frac{\partial F_{1}}{\partial x_{0}} & \frac{\partial F_{1}}{\partial y_{0}} & \frac{\partial F_{1}}{\partial \theta }\\ \ldots & \ldots & \ldots & \ldots & \ldots \\ \frac{\partial {\vec{F}}_{n}}{\partial a} & \frac{\partial {\vec{F}}_{n}}{\partial b} & \frac{\partial {\vec{F}}_{n}}{\partial x_{0}} & \frac{\partial {\vec{F}}_{n}}{\partial y_{0}} & \frac{\partial {\vec{F}}_{n}}{\partial \theta }\\ 0 & 0 & 2\lambda x_{0} & 2\lambda y_{0} & 0 \end{array}\right]\left[\begin{array}{c} \Delta a\\ \Delta b\\ \Delta x_{0}\\ \Delta y_{0}\\ \Delta \theta \end{array}\right]=\left[\begin{array}{c} {d_{1}}^{2}-F_{1}\\ \ldots \\ {d_{n}}^{2}-F_{n}\\ \lambda \left[0-\left(x_{0}^{2}+y_{0}^{2}\right)\right] \end{array}\right].$$

Then we define $F_{i}$ as

(14) $$F_{i}=\frac{{\left(\cos\theta \cdot dx_{i}+\sin\theta \cdot dy_{i}\right)}^{2}}{a^{2}}+\frac{{\left(-\sin\theta \cdot dx_{i}+\cos\theta \cdot dy_{i}\right)}^{2}}{b^{2}},$$

and $e_{i}\left\{i=1,2\ldots n\right\}$ is the intensity. We compute the params $\left\{a,b,x_{0},y_{0},\theta \right\}$ by minimizing the Mahalanobis distance:

(15) $$\min_{a,b,x_{0},y_{0},\theta }\left[\sum_{i=1}^{n}{\left(d_{i}-F_{i}\right)}^{T}\left(d_{i}-F_{i}\right)+e^{-2}\lambda^{2}{\left(x_{0}^{2}+y_{0}^{2}\right)}^{2}\right].$$

In addition, if an object has no direction, the ellipse fitting equation is defined as

(16) $$\frac{{\left(x_{i}-x_{0}\right)}^{2}}{a^{2}}+\frac{{\left(y_{i}-y_{0}\right)}^{2}}{b^{2}}=d_{i}^{2}.$$

## 4. Experiments

### 4.1. Datasets

For experiments, we choose three datasets, known as DOTA, MS COCO, and PASCAL VOC for object detection.

DOTA [25,29]. It is the largest dataset for object detection in aerial images with oriented bounding box annotations. It contains 2806 large size images. There 15 categories, including Baseball diamond (BD), Ground track field (GTF), Small vehicle (SV), Large vehicle (LV), Tennis court (TC), Basketball court (BC), Storage tank (ST), Soccer-ball field (SBF), Roundabout(RA), Swimming pool (SP), and Helicopter (HC) [25,29]. The fully annotated DOTA images contain 188, 282 instances. We cut these images into subgraphs of size 416×416 and use these subgraphs as a collection of training samples.

MS COCO [27]. MS COCO is a large-scale object detection, segmentation, and captioning dataset. We used MS COCO 2014 dataset in our experiment. It contains 80 k training images, 40 k validation images and 40 k testing images.

PASCAL VOC [26]. The PASCAL Visual Object Classes is a world-class computer vision challenge that has emerged with many classic object detection and segmentation models. The most widely used datasets are VOC 2007 and VOC 2012. The VOC 2007 dataset consists of about 5k trainval images and 5 k test images over 20 object categories [2]. And the VOC 2012 has 11 k trainval images. In order to increase the amount of data, we combine these two datasets and then experiment based on this.

### 4.2. Implementation Details

We use the Darknet [1] framework for all training and inference. Darknet is an open source neural network framework written in C and CUDA. It is fast, easy to install, and supports CPU and GPU computation [30]. The classic object detection algorithm Yolo [1,9,10] is based on Darknet.

In the experiments, we trained two basic field models, U-Field-Net and FCN-Field, using U-Net and FCN as backbone respectively. For training, we firstly set up encode-decode structure network to construct FCN-Field. Then we use the route layer to concat the output of upsample layer in the network decode part and the same size layer before maxpooling in the encode layer. Then the structure is constructed. And the batch size of FN is set to 8, the learning rate is set to 0.00001 for the first. Then it will be dropped by 10% at 100 and 50,000 batches respectively. The input image is resized to 224×224.

### 4.3. Ablation Studies

We conduct a serial of ablation experiments on DOTA to find the appropriate settings of our proposed FN. And we use the U-Net and FCN as our baseline respectively. Then gradually change the settings. Table 1 summarizes the results of ablation studies at the training. It can be seen from the table that the U-Field-Net mAP is significantly higher than the Field-FCN. This is because the cross-connect between the encode and decode layers improves the ability to express network features. In addition, the mAP model with the batch normalize layer is higher overall. As described in Reference [9], batch normalize is a good way to prevent overfitting. We found that the larger the batch each subdivision, the better the model obtained, but at the cost of more memory resources consumed. The batch size is set to 64 which can get better performance under the same resolution, which is consistent with the configuration in Reference [9].

The output layer of U-Field-Net contains 2×C feature maps. If there are more categories, the model will be larger, so we designed a simplified model, as shown in the last row of Table 1. From this group of experiments, we can see that the highest accuracy can be achieved when batch normalize, batchsize = 64 and subdivision = 8 are enabled. We combine 2×C output fields into 2, and add a group of softmax layers composed of C+1 output feature maps, so that the feature maps of the output layer are reduced from 2×C to C+3.

At the inference phase, we also did ablation studies. Table 2 shows the results of the experiments according to Equation (13). We use hit precision (HP) to describe the accuracy of object detection at the inference. HP is defined as follows:

(17) $$HP=\frac{TP}{TP+FP+TN}.$$

It can be seen from Table 2 that at inference, using Ransac, scaling the local graph and using the central constraint to solve the object elliptic equation can achieve the highest accuracy, which is significantly better than other methods not fully adopted.

In order to study the influence of tensor obj transform on the model, we compared several typical backbones in anchors, points and FN. The representatives of anchors are Yolo [10] and RoI Transform [29]; the representative algorithm of points is CenterNet [31]. Table 3 and Table 4 show the mAP comparison between our FN method and other methods under the same backbone on VOC and DOTA. It can be seen from the table that the accuracy of FN method is significantly higher than that of other methods.

### 4.4. Comparison with the State-of-the-Art Methods

We compared the performance of our proposed FN with the state-of-the-art algorithms on three datasets DOTA [25], MS COCO [27] and PASCAL VOC [26]. Yolo, SSD and Retinanet are all one stage algorithms, and anchors are used for regression. Faster-Rcnn is a two stage algorithm, which adopts anchors for RPN regression. Cornernet is the anchor free method, which uses the corner points in the upper left corner and the lower right corner to predict bbox. Different from the above methods, our method has the function of object detection and direction judgment through the regression of object field.

**Figure 7:**
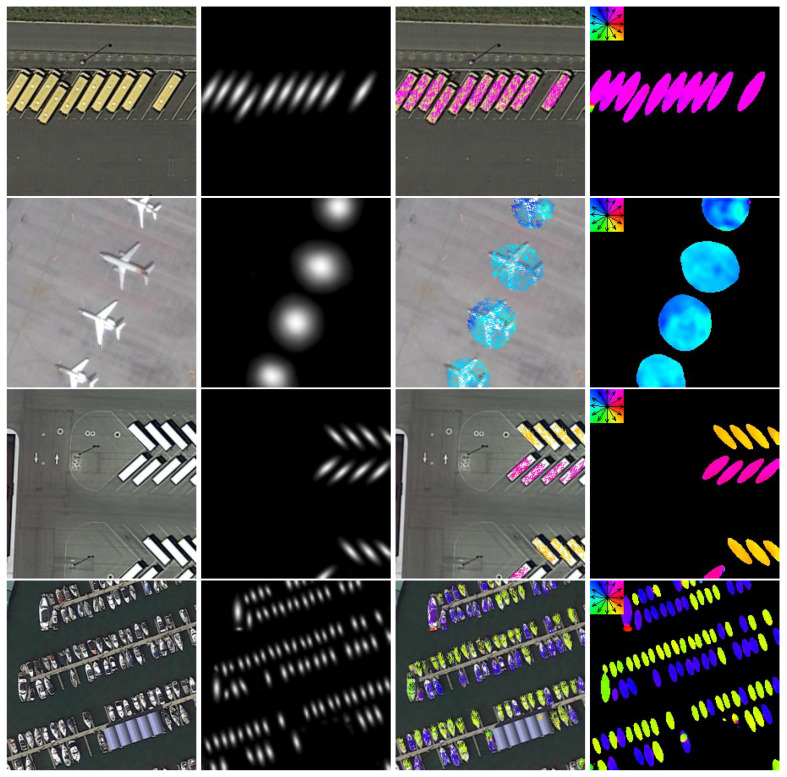
Visualizations of the Field’s results on the DOTA dataset. The first column is the original image overlying with the object ellipse and its bounding rectangle, the second column is the Center Field, the third column is the direction diagram and the last column is the Direction Field.

**Figure 8:**
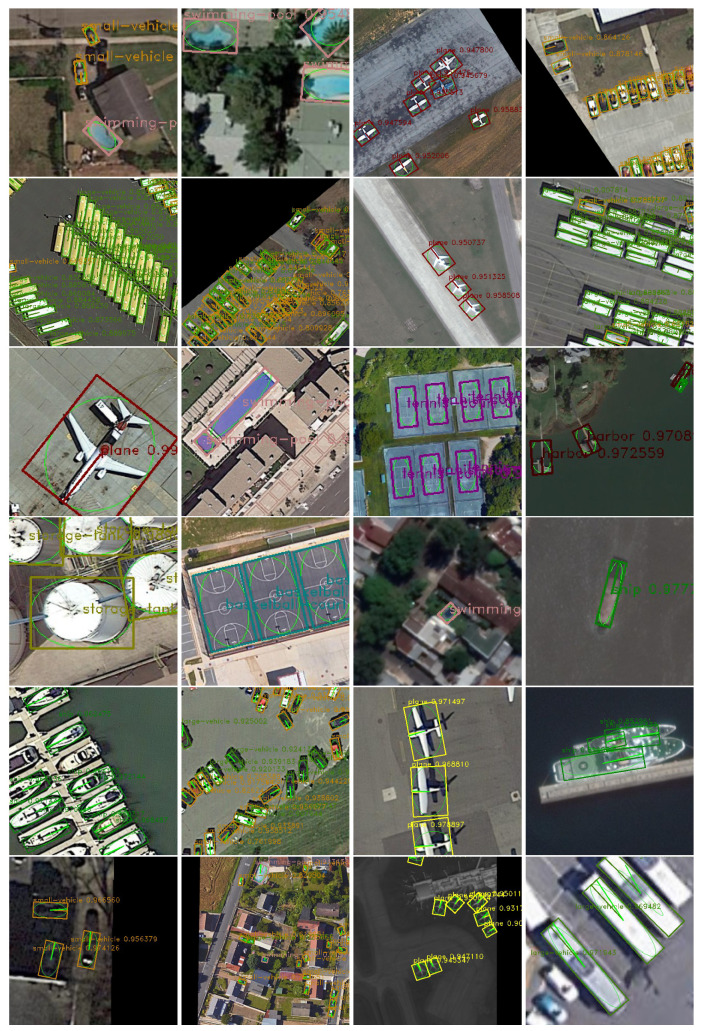
Visualizations of results on the DOTA dataset. The line segment starting from the object center point identifies the direction of the object. The category object marked with a main direction line in the area of the object oblique quadrilateral is the object type marked with * in Table 5.

**Performance on the DOTA dataset.** In Table 5, we compare our method with state-of-the-art detectors on the DOTA dataset. As can be seen from this table, FN based on Field-FCN achieved the mAP of 74.74 for DOTA, it outperforms the previous RoI Trans(69.56) by 5.18 points. Furthermore, FN based on U-Field-Net also achieved the mAP of 75.18, which has improvement by 0.71 points. We give some qualitative results of FN on DOTA in Figure 7 and Figure 8. The direction error is shown in Table 6. The previous methods can only find the quadrilateral of the object, but not the direction. Therefore, we give the accuracy index of the direction vector of the remote sensing objects.

**Performance on the MS COCO dataset.** In Table 7, we compare our method with that of References [10,39,40] on the MS COCO dataset. Our method achieves the state-of-the-art performance on mAP. Specifically, based on our proposed method, the mAP can reach 61.2, which is the best performance among these methods.

**Performance on the PASCAL VOC dataset.** In Table 8, we also compare our method with the state-of-the-art methods on PASCAL VOC dataset. From the table we can see that our method also achieves the best performance, which is 82.0.

### 4.5. Running Time

Given a 224×224 image, our method runs at 25 fps on a desktop with an Intel E5 3.5GHz CPU and a RTX 2080Ti GPU, which is efficient for real-time object detection. Table 9 shows the performance of FN. As we can see from the table, our model is only 73M. At the same time, when the input image size is 576×576, our model detection time only needs 0.1s to achieve the mAP of 75.35.

## 5. Conclusions

In this paper, we proposed an algorithm based on a field—called FN—for object detection, which can effectively balance speed and accuracy. The field can reflect the intensity of the object area. Our algorithm can not only detect the objects, but also determine the direction. Moreover, even if it is a big image, we can detect it by spray painting without cutting. Compared with the traditional ROI method, our method can describe the geometric distribution of the object space more accurately. At the same time, the directional field regression method proposed in this paper can be used to study the output direction field of directional object categories (such as aircraft, ship, car). In the future, we will consider using this method to achieve a probabilistic and directional semantic segmentation, and increase the probability and direction information based on the segmentation algorithm to improve the ability to understand the scene semantics. This method can be applied to many computer vision applications. Furthermore, we reported the state-of-the-art performances on three widely-used datasets and demonstrated the rationality of the proposed approach. The method proposed in this paper is not limited to air image for any scene image. And the ground objects captured by satellite images have significant two-dimensional directivity, which is convenient for us to carry out experimental tests. Also, our method has some limitations. When the input image resolution is large, the learning speed is slow, and a larger backbone is needed.

## Figures and Tables

**Table 1:** Results of ablation studies on the DOTA dataset at the training. We built 8 models by adding batch normalize to the convolutional layers and using different amounts of batch size in the Darknet.

Method	Batch Normalize	Batch Size	Subdivisions	Batch Each Subdivision	mAP
FCN-Field	✓	64	8	8	74.74
U-Field-Net	✓	64	8	8	**75.18**
	✓	16	4	4	75.05
	✓	8	1	8	75.12
	✓	4	1	1	74.97
		64	8	8	74.78
		16	4	4	74.56
		4	1	4	74.52
		8	1	8	74.76
U-Field-Net-1C	✓	64	8	8	63.72

**Table 2:** Results of ablation Algorithm (RFA) on the DOTA dataset at the training. Ransac means whether to use the ransac method when calculating ellipse parameters. Resize indicates whether the output field is enlarged by 2 times. Central restraint condition means whether to consider the central constraint by Equation (12).

Method	Ransac	Resize	Central Restraint Condition	HP
U-Field-Net	✓	✓	✓	**78.43**
		✓	✓	73.56
	✓		✓	73.14
	✓	✓		73.43
	Replaced by min AreaBox	✓	✓	73.15

**Table 3:** Comparisons with different backbones on VOC dataset.

Backbone	Anchors	Points	FN
VGG16 [32]	77.65	73.37	79.92
FPN [15]	79.82	76.82	81.36
Hourglrass [21]	79.64	76.05	80.25
DLA [33]	78.27	75.98	79.68

**Table 4:** Comparisons with different backbones on DOTA dataset.

Backbone	Anchors	Points	FN
VGG16 [32]	67.74	61.37	73.52
FPN [15]	69.56	63.16	75.18
Hourglrass [21]	68.96	63.04	75.17
DLA [33]	68.37	62.98	75.03

**Table 5:** Comparisons with state-of-the-art detectors on DOTA [25]. The short names for each category can be found in Reference [29]. The object class marked with * has a directional attribute, that is $w_{c}=1$ in the Equation (6). Selective regression of the directional fields of these categories can significantly improve accuracy.

Method	Plane *	BD	Bridge	GTF	SV *	LV *	Ship *	TC	BC	ST	SBF	RA	Harbor *	SP	HC *	mAP
FR-O [25,29]	79.42	77.13	17.7	64.05	35.3	38.02	37.16	89.41	69.64	59.28	50.3	52.91	47.89	47.4	46.3	54.13
RRPN [34]	80.94	65.75	35.34	67.44	59.92	50.91	55.81	90.67	66.92	72.39	55.06	52.23	55.14	53.35	48.22	60.67
R2CNN [29,35]	88.52	71.2	31.66	59.3	51.85	56.19	57.25	90.81	72.84	67.38	56.69	52.84	53.08	51.94	**53.58**	61.01
R-DFPN [36]	80.92	65.82	33.77	58.94	55.77	50.94	54.78	90.33	66.34	68.66	48.73	51.76	55.1	51.32	35.88	57.94
Yang et al. [29,37]	81.25	71.41	36.53	67.44	61.66	50.91	56.6	90.67	68.09	72.39	55.06	55.6	62.44	53.35	51.47	62.29
ICN [38]	81.36	74.3	**47.7**	70.32	64.89	67.82	69.98	90.76	79.06	78.2	53.64	**62.9**	**67.02**	64.17	50.23	68.16
ROI Trans. [29]	88.64	**78.52**	43.44	75.92	68.81	73.68	83.59	90.74	77.27	**81.46**	58.39	53.54	62.83	58.93	47.67	69.56
FCN-Field	93.06	76.21	36.93	78.24	88.25	89.02	90.18	90.75	**95.37**	77.82	62.47	56.98	40.02	94.37	51.41	74.74
U-Field-Net	**93.45**	76.41	37.04	**78.54**	**88.71**	**89.94**	**90.60**	**90.90**	95.24	78.16	**62.81**	57.43	40.69	**95.40**	52.39	**75.18**

**Table 6:** Direction error on DOTA. $\lambda_{d}$ is defined in Equation (2). Error with * indicates that we regress the Direction Field of the objects with * in the Table 5. Error with all means that we regress the Direction Field of all objects. Experiments show that for some objects that have no direction, or symmetric objects, such as storage tanks, direction regression will reduce the accuracy of overall direction prediction.

Parameter	$\lambda_{d}=2$	$\lambda_{d}=10$	$\lambda_{d}=20$
Error with * (degree)	8.5	4.1	2.3
Error with all (degree)	10.6	6.2	4.0
Percentage within 10 degrees	48.5	92.7	96.1

**Table 7:** Comparisons with the state-of-the-art methods on MS COCO. The YO-v3 indicates the YOLOv3 method. The Re-Net indicates the RetinaNet method and the Cor means the CornerNet method.

Method	YO-v3 [10]	Re-Net [39]	Cor [40]	OURS
mAP	57.9	61.1	56.5	**61.2**

**Table 8:** Comparisons with the state-of-the-art methods on PASCAL VOC. The YO-v3 indicates the YOLOv3 method. The F-RCN means the Faster RCNN  method.

Method	YO-v3 [10]	F-RCN [2]	SSD [3]	OURS
mAP	63.4	70.0	76.8	**79.1**

**Table 9:** Performance testing of our U-Field-Net (including post process time) on DOTA. All the speed are tested on a single RTX 2080Ti.

Input Size	mAP	Test Speed	Param	BFLOPs
224×224	75.18	0.025s	73M	24.31Bn
448×448	75.32	0.051s	73M	97.23Bn
576×576	75.35	0.106s	73M	198.56Bn

## Abbreviations

The following abbreviations are used in this manuscript:

MDPI	Multidisciplinary Digital Publishing Institute
DOAJ	Directory of open access journals
TLA	Three letter acronym
LD	linear dichroism
